# Evidence of accelerated epigenetic aging of breast tissues in patients with breast cancer is driven by CpGs associated with polycomb-related genes

**DOI:** 10.1186/s13148-022-01249-z

**Published:** 2022-02-24

**Authors:** Mariya Rozenblit, Erin Hofstatter, Zuyun Liu, Tess O’Meara, Anna Maria Storniolo, Disha Dalela, Vineet Singh, Lajos Pusztai, Morgan Levine

**Affiliations:** 1 Department of Internal Medicine, Section of Medical Oncology, Yale School of Medicine, 300 George Street, Suite 120, New Haven, CT 06511 USA; 2 Department of Big Data in Health Science, School of Public Health and Center for Clinical Big Data and Analytics, Second Affiliated Hospital, Zhejiang University School of Medicine, Hangzhou, Zhejiang China; 3 Department of Internal Medicine, Indiana University Melvin and Bren Simon Cancer Center, Indianapolis, IN 46202 USA; 4 Department of Pathology, Yale School of Medicine, 330 Cedar Street, New Haven, CT 06511 USA

**Keywords:** Breast cancer, Epigenetic clocks, Methylation changes, Polycomb-related genes

## Abstract

**Purpose:**

Age is one of the strongest risk factors for the development of breast cancer, however, the underlying etiology linking age and breast cancer remains unclear. We have previously observed links between epigenetic aging signatures in breast/tumor tissue and breast cancer risk/prevalence. However, these DNA methylation-based aging biomarkers capture diverse epigenetic phenomena and it is not known to what degree they relate to breast cancer risk, and/or progression.

**Methods:**

Using six epigenetic clocks, we analyzed whether they distinguish normal breast tissue adjacent to tumor (cases) vs normal breast tissue from healthy controls (controls).

**Results:**

The Levine (*p* = 0.0037) and Yang clocks (*p* = 0.023) showed significant epigenetic age acceleration in cases vs controls in breast tissue. We observed that much of the difference between cases and controls is driven by CpGs associated with polycomb-related genes. Thus, we developed a new score utilizing only CpGs associated with polycomb-related genes and demonstrated that it robustly captured epigenetic age acceleration in cases vs controls (*p* = 0.00012). Finally, we tested whether this same signal could be seen in peripheral blood. We observed no difference in cases vs. controls and no correlation between matched tissue/blood samples, suggesting that peripheral blood is not a good surrogate marker for epigenetic age acceleration.

**Conclusions:**

Moving forward, it will be critical for studies to elucidate whether epigenetic age acceleration in breast tissue precedes breast cancer diagnosis and whether methylation changes at CpGs associated with polycomb-related genes can be used to assess the risk of developing breast cancer among unaffected individuals.

**Supplementary Information:**

The online version contains supplementary material available at 10.1186/s13148-022-01249-z.

## Introduction

Outside of genetic mutations in BRCA genes, age is the strongest risk factor for the development of breast cancer. However, it remains unclear whether the link between age and breast cancer is due to a shared underlying etiology, or instead, a probabilistic relationship in which the risk of acquiring cancer-driving mutations in tissues increases as a function of chronological time. Providing some evidence for the potential of shared mechanism, it has been noted that many of the known hallmarks of aging are also hallmarks in cancer. One hallmark with considerable overlap is epigenetic alterations—or more specifically, changes in DNA methylation (DNAm). DNAm typically refers to the addition of a methyl group (CH3) to a CpG dinucleotide (5’—C—phosphate—G—3’) [1]. DNAm impacts transcriptional repression/activation and is thought to control a number of cellular properties from differentiation to replication. Interestingly, both cancer and aging have been associated with specific changes in the pattern of DNAm, often characterized by gains of DNAm at gene promotors and loss of global DNAm, particularly in intergenic regions associated with dispersed retrotransposons, heterochromatic DNA repeats, and endogenous retroviral elements [2].

Given that DNAm levels at specific CpGs have been shown to predictably change with age [3–7] our group and others have developed measures that can estimate the age of a tissue or biofluid sample from DNAm data. These aging biomarkers, referred to as “epigenetic clocks” [8–11], strongly correlate with chronological age in multiple tissues, but more importantly, it has been shown that the discordance between observed age and predicted age holds biological meaning—epigenetic age acceleration (defined as a predicted epigenetic age that exceeds chronological age) has been associated with several health conditions, including obesity [12], cancer [13–16] and all-cause mortality [17–21]. We have also previously shown that malignant breast cancer samples from luminal breast cancer exhibit increased epigenetic age acceleration. In addition, these positive epigenetic age accelerations are seen in normal breast tissue that is adjacent to breast tumor [22]. One hypothesis is that aging tissues, including breast, may accumulate epigenetic changes that predispose them to tumorigenesis. If true, elucidating the underlying biology that connects aging and cancer may facilitate primary and secondary prevention and/or treatment.

While our prior study utilized one of the earliest developed epigenetic clocks, known as the Horvath clock, there are more than a dozen epigenetic clocks that have been developed to date. Each clock uses slightly different methods for estimating epigenetic age. They incorporate different numbers and sets of CpGs, and when being developed, they relied on different types of samples, including whole blood, saliva, and/or tissues. As a result, the various clocks exhibit different degrees of association with either chronological age or various health outcomes, like cancer [23], suggesting that they may capture different aspects of the biological aging process. Furthermore, our work has shown that biological clocks are composite measures that capture signals from diverse types of DNA methylation changes. Thus, when it comes to links between epigenetic aging and outcomes, like breast cancer, it is not clear whether the associations stem from multiple facets of the epigenetic clocks, or instead, from a few distinct types of epigenetic age changes. Lastly, it also remains unknown whether the aging changes related to cancer risk in a given tissue are observable in easily accessible biofluids, like blood.

Thus, the goal of this study was to identify which epigenetic clock signatures can distinguish tumor-adjacent breast tissue from cancer patients versus normal tissue from healthy controls. We focused on six epigenetic clocks that are the most utilized in the literature. We also examined concordance between epigenetic age assessment from paired breast tissue and peripheral blood to assess whether blood could serve as a reliable and non-invasive proxy for epigenetic aging in breast.

## Methods

### Study specimens

Study specimens were used from a previous study [22] which included four cohorts; 1) DNA collected from peripheral blood from breast cancer patients (*n* = 79) presenting to Yale New Haven Hospital with a new diagnosis of breast cancer who had not received any chemotherapy, radiation, or endocrine therapy prior to surgery (cases), 2) DNA collected from peripheral blood from women without cancer (*n* = 91) from the Susan G. Komen Tissue Bank at IU Simon Cancer Center and women presenting for reduction mammoplasty at Yale New Haven Hospital (controls), 3) fresh frozen normal appearing breast tissue (*n* = 34) at least 3 cm away from the primary tumor margin from breast cancer patients presenting to Yale New Haven Hospital with a new diagnosis of breast cancer who had not received any chemotherapy, radiation, or endocrine therapy prior to surgery (cases), 4) fresh frozen normal breast tissue (*n* = 50) from women without cancer from the Susan G. Komen Tissue Bank at IU Simon Cancer Center and women presenting for reduction mammoplasty at Yale New Haven Hospital (controls). Clinical data collected for each participant included age, body mass index, race, ethnicity, medical history, reproductive history, tobacco and alcohol use, family history of breast cancer, and tumor characteristics. The study was approved by the institutional review board, and written informed consent was obtained from all patients.

### Tissue processing and DNA extraction

Breast tissue was processed as previously described [22]. Briefly, six individual tissue cores were placed in 10% buffered formalin solution and flash frozen with liquid nitrogen. DNA was extracted from at least 50 mg of tissue using the Qiagen All Prep Universal Kit [cat. 80224]. Whole blood samples were collected at the time of surgery (for mastectomy and reduction mammoplasty patients) or initial Breast Cancer Prevention Clinic intake and stored at -80℃. DNA was then extracted from 200 µL of whole blood using the Qiagen DNA Mini Blood Mini Kit [cat. 51104]. DNA received from KTB was reconstituted in TE buffer. 1 µg of extracted DNA was sent for bisulfite sequencing experiments.

### Methylation studies

Zymo EZ DNA methylation KIT (Zymo Research Orange CA USA) was used to obtain bisulfate conversion and subsequent hybridization, and scanning was performed with the HumanMethylation450k BeadChip (Illumina, San Diego, CA) and iScan (Illumina) according to the manufacturer’s protocol with standard settings. DNA methylation levels were quantified using the “noob” normalization method [24]. Specifically, the β value was calculated as a ratio of the intensity of fluorescent signals from the methylated and the unmethylated sites:

$$\upbeta = {\text{max}}\left( {{\text{M}},0} \right)/\left[ {{\text{max}}\left( {{\text{M}},0} \right) + {\text{max}}\left( {{\text{U}},0} \right) + {1}00} \right].$$

$$\begin{aligned} & {\text{M}} = {\text{methylated}}\,{\text{signals}}. \\ & {\text{U}} = {\text{unmethylated}}\,{\text{signal}}. \\ \end{aligned}$$

Thus, β values ranged from 0 to 1 (completely unmethylated to completely methylated).

### Epigenetic variables

Using the DNAm beta values from both breast tissue and whole blood, DNAmAge was then calculated for six clocks in accordance with previously published algorithms [23]. For instance, each clock is based on a different set of CpGs, for which a weighted sum is used to designate epigenetic age.

### Statistical methods

Baseline patient characteristics were compared in the cancer and control arm to identify any differences in the study cohort. Biweight midcorrelation was used to assess that association between two continuous variables, for instance, between epigenetic clocks and chronological age, and/or paired epigenetic clock values (between breast and blood or two different clocks). Epigenetic clock scores were also estimated that adjusted out the effect of covariates, such as age, race/ethnicity, BMI, smoking status, alcohol use, menopausal status, OCP use, and HRT use. These adjusted clock scores—termed “epigenetic age acceleration”—were based on residuals from OLS regression models in which epigenetic age is regressed on confounders. These epigenetic age scores were then used to test for differences between cases and controls via Kruskal–Wallis test.

## Results

### Patient demographics

Normal breast tissue (> 3 cm away from breast tumor) was analyzed from 34 patients with breast cancer (cases) and 50 healthy controls (controls), and peripheral blood samples were analyzed from 79 patients with breast cancer and 91 healthy controls. Matched breast and blood samples were available from 80 patients (Additional file 1: Fig. S1). The characteristics of the study population are summarized in Table 1. Overall, the breast cancer and control cohorts were very similar. The mean age of the breast cancer cohort was 51.4 years, and the mean age of the control cohort was 47.6 years. Both cohorts included mostly non-Hispanic white patients (75% in the breast cancer cohort, and 88% in the control cohort), with similar BMIs (27.4 in the breast cancer cohort, and 27.7 in the control cohort), mostly non-smokers (57.5% in the breast cancer cohort, and 61.7% in the control cohort), with some alcohol consumption (65.5% in the breast cancer cohort, and 95.7% in the control cohort). The majority of cancers were hormone positive (63.7%), HER2 negative (72.6%), and BRCA negative (57.5%).

**Table 1:** Characteristics of the study population

Characteristics	Tissue sample		Blood sample	
	Breast cancer *N* = 34	Control *N* = 50	Breast cancer *N* = 79	Control *N* = 91
Age, years	48.3 ± 12.2	46.7 ± 20.8	54.6 ± 13.6	48.5 ± 17.7
Cohort (Komen/Yale)	0/34	45/5	0/79	44/47
Age groups				
< 50 years	21	27	32	44
≥ 50 years	13	23	47	47
Race/ethnicity				
Non-Hispanic White	23	45	62	79
Hispanic White	3	2	3	4
Africa American	4	3	7	6
Others	4	0	7	2
Body mass index	26.6 ± 4.9	26.9 ± 5.3	28.2 ± 6.1	28.5 ± 6.7
Smoking				
Non-smoker	21	31	44	56
Former smoker	12	14	30	25
Current smoker	1	5	5	10
Alcohol consumption				
No	18	3	21	3
Yes	16	47	58	88
Age at first birth	25.5 ± 5.6	25.8 ± 3.4	26.0 ± 5.0	26.2 ± 4.5
Menopause, yes				
No	24	25	35	37
Yes	10	25	44	54
Age at menses onset	12.3 ± 1.7	12.5 ± 1.1	12.7 ± 1.7	12.4 ± 1.4
Number of birth	1.8 ± 1.4	2.2 ± 0.6	1.9 ± 1.4	2.1 ± 1.0
Total pregnancy	2.3 ± 1.9	2.5 ± 1.0	2.3 ± 1.9	2.6 ± 1.5
ER/PR				
+/+	23	−	49	−
±	1	−	2	−
−/−	1	−	10	−
Unknown	9	−	18	−
HER2				
Positive	3	−	4	−
Negative	24	−	58	−
Not typed/insufficient tissuee	7	−	10	−
Unknown	0	−	7	−
BRCA testing				
Positive	2	−	2	0
Negative	16	−	49	33
Results unknown	6	−	12	0
Not tested	10	−	16	9
Oral contraceptive pill use				
Current and former user	5	3	26	36
Never	13	0	23	8
Unknown	16	47	30	47
Ever hormone replacement therapy				
Yes	3	45	12	20
No	3`	35	67	71

### Epigenetic age in normal breast tissue of breast cancer patients and controls

**Fig. 1:**
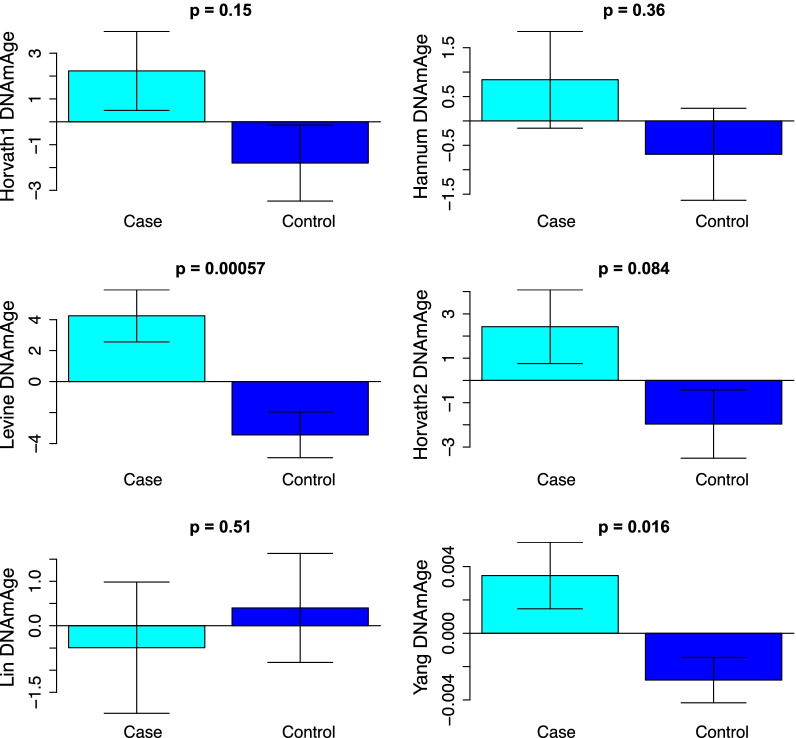
Epigenetic clock accelerations in cases (normal breast tissue > 3 cm away from breast tumor in breast cancer patients) and controls (normal breast tissue from healthy individuals) adjusted for chronological age

The correlation between epigenetic clocks (measured in breast tissue) and chronological age was examined for both cases and controls. When examining epigenetic aging in control samples, significant age correlations were found for five of the six clocks, ranging from *r* = 0.35 (Levine clock) to *r* = 0.68 (Lin clock), while the Yang clock did not exhibit a significant increase in epigenetic age as a function of chronological age in controls. On the other hand, no significant age correlations were found for any of the clocks when restricting samples to cases only (Additional file 1: Fig. S2), suggesting that chronological age did not differentiate epigenetic ages in normal breast tissue scores among women who had already developed breast cancer. When comparing epigenetic ages of cases to controls, we found that increased epigenetic age acceleration (age adjusted) was observed for the Levine (*p* = 5.7e−4) and Yang (*p* = 1.6e−2) clocks in the normal breast tissue from breast cancer patients (cases) compared to controls (Fig. 1). These results were maintained even after adjusting for the full list of confounders in our data (Additional file 1: Fig. S3).

**Fig. 2:**
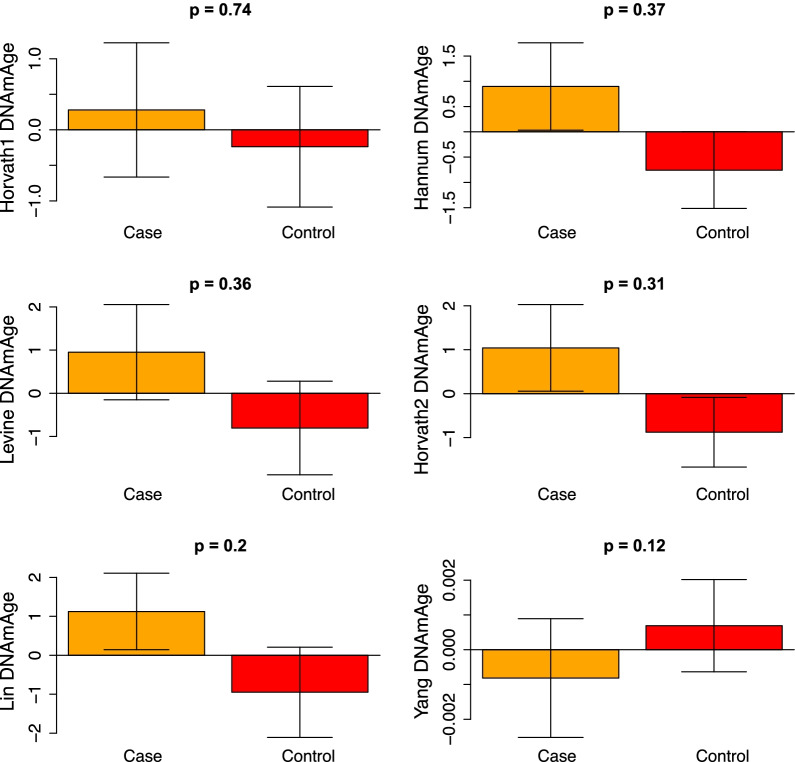
Epigenetic clock accelerations in peripheral blood from cases (breast cancer patients) and controls (healthy individuals) adjusted for chronological age

In the peripheral blood, all six epigenetic clocks correlated with age in both cases and controls, and we observed much higher age correlations than were observed in breast tissue (Additional file 1: Fig. S4). For instance, age correlations in controls ranged from *r* = 0.43 (Yang) to *r* = 0.87 (Horvath2). Similarly, age correlations in cases ranged from *r* = 0.26 (Yang) to *r* = 0.84 (Hannum and Horvath2). However, when comparing epigenetic age acceleration between cases and controls, no clocks showed statistically significant differences (Fig. 2).

**Fig. 3:**
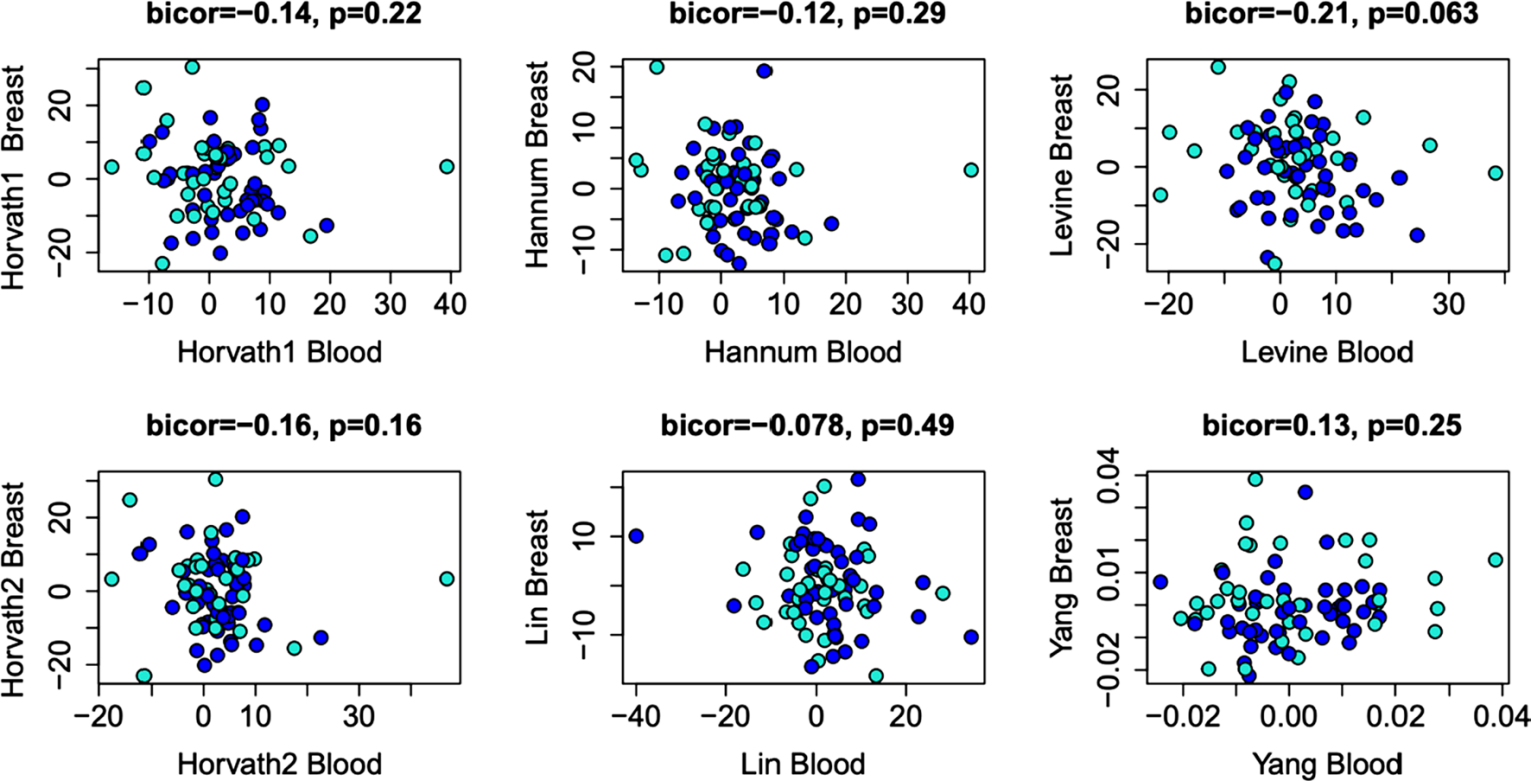
Correlations between epigenetic age in matched samples of peripheral blood and breast tissue using six different epigenetic clocks

Finally, we compared the predicted epigenetic age acceleration values in breast tissue vs. peripheral blood using matched samples. Results showed no significant associations across any of the six clocks (Fig. 3). This suggests that aging in the breast tissue is likely discordant with aging in the blood and, therefore, peripheral blood would not serve as a useful surrogate measure when it comes to assessing the epigenetic age in breast tissue.

### Epigenetic age acceleration seen in normal breast tissue from breast cancer patients is driven by polycomb-related genes

The Levine and Yang clocks that were associated with breast cancer and demonstrated significant age acceleration share a characteristic in that they are both enriched for CpGs associated with polycomb group (PcG) protein targets. For instance, the Yang clock was intentionally developed to comprise only CpGs found in PcG regions of the genome, while the Levine clock had no CpG preselection, but was found to be enriched for PcG regions, particularly among its CpGs that exhibit hypermethylation age. Given that the Levine clock (1) showed the strongest association with case versus control status, and (2) is comprised of CpGs that both are and are not associated with PcG target genes, we set to test whether the associations with breast cancer differed between the signal coming from PcG-related CpGs versus the remaining (non-PcG) CpGs. To do this, the Levine clock score was recalculated twice, but in each case, CpGs belonging to either PcG- or non-PcG-related regions were set to zero, such that they dropped out of the model. This left us with two clocks scores that together sum to the full Levine clock score, yet enabled us to test the associations between breast cancer and these two distinct types of epigenetic age changes. CpGs associated with polycomb-related genes were defined as regions with binding sites of SUZ12 [25].

**Fig. 4:**
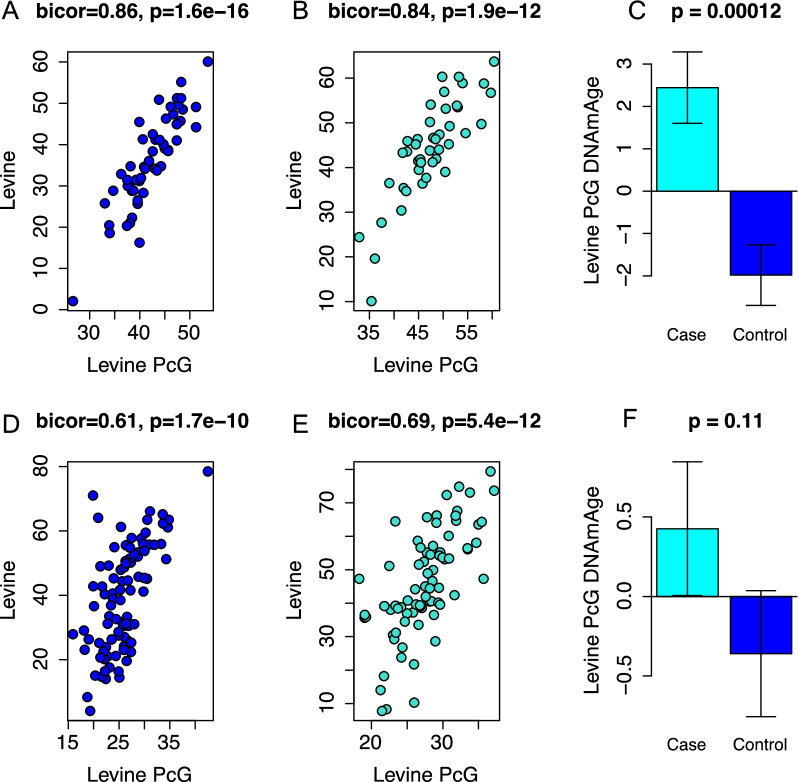
Epigenetic age acceleration using only CpGs associated with polycomb-related genes in the Levine clock. Correlation between the overall Levine clock and the polycomb-related genes score in controls (**A**), cases (**B**) and cases vs controls (**C**) in breast tissue (top row) and correlations between the overall Levine clock and polycomb-related genes score in controls (**D**), cases (**E**), and cases vs controls (**F**) in peripheral blood (bottom row)

When only the CpGs associated with polycomb-related genes (*n* = 55) from the Levine clock are used, we found that the score was still highly correlated with the original Levine clock score in both cases and controls. Interestingly, this correlation is higher in breast tissue for both controls (*r* = 0.86, Fig. 4A) and cases (*r* = 0.84, Fig. 4B) than it is in peripheral blood (Fig. 4D, E). Furthermore, this aging score based on CpGs that are associated with only PcG-related genes has an even higher association with cases than controls in breast tissue (*p* = 0.00012) compared to the entire Levine clock (Fig. 4C).

**Fig. 5:**
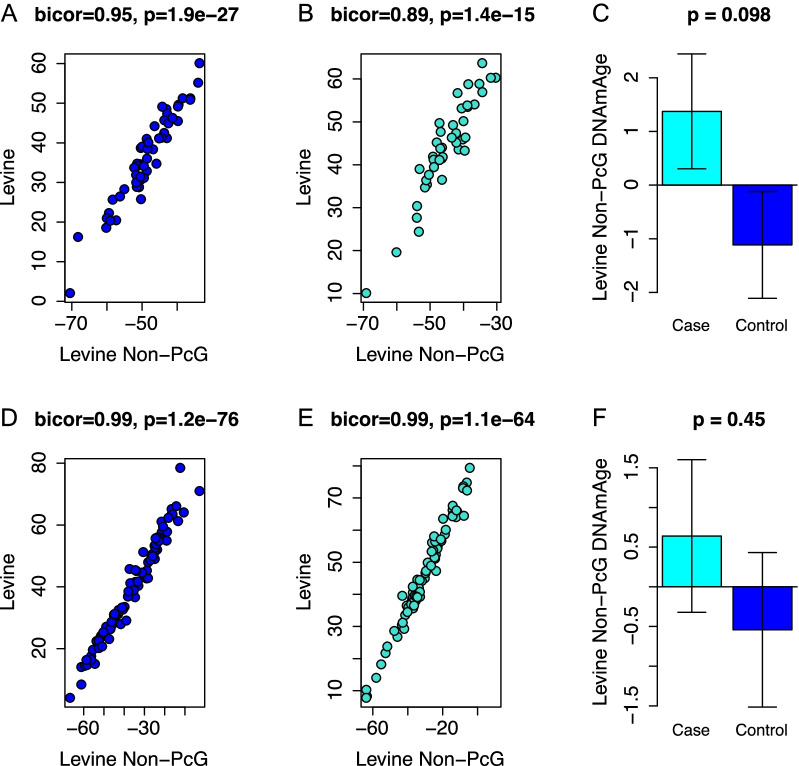
Epigenetic age acceleration using only CpGs not associated with polycomb-related genes in the Levine clock. Correlation between the overall Levine clock and the polycomb-related genes score in controls (**A**), cases (**B**) and cases vs controls (**C**) in breast tissue (top row), and correlations between the overall Levine clock and polycomb-related genes in controls (**D**), cases (**E**), and cases vs controls (**F**) in peripheral blood (bottom row)

**Fig. 6:**
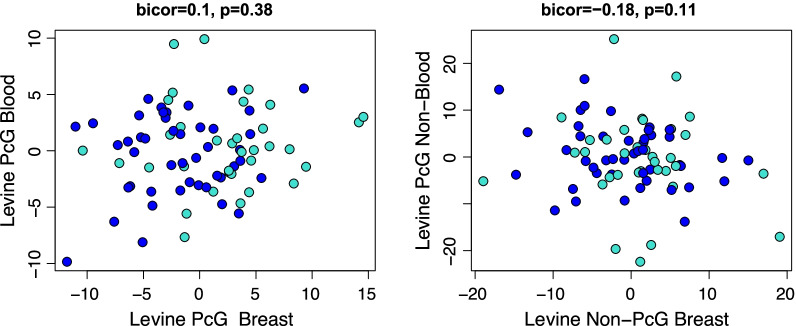
Correlations between CpGs associated with polycomb-related genes (left) and CpGs not associated with polycomb-related genes (right) between matched samples in peripheral blood and breast tissue

When calculating the epigenetic clock using the CpGs that are not associated with PcG genes (*n* = 458), we find that these scores are highly correlated with the Levine clock scores in both breast and peripheral blood of cases and controls (range *r* = 0.89 to *r* = 0.99). However, we do not observe a significant difference between cases and controls (Fig. 5C, F). Finally, when comparing the breast vs. blood concordance in matched samples for these two subclocks, again, no significant association is found, suggesting blood and breast may be aging asynchronously (Fig. 6).

## Discussion

Our analysis suggests that methylation signals in breast tissues captured by epigenetic clocks are strongly associated with breast cancer status. Two clocks, Levine and Yang, are significant and show epigenetic age acceleration in normal breast tissue that is adjacent to breast tumors compared to normal tissue from healthy controls. All epigenetic clocks were highly correlated with age in controls, suggesting that the CpGs that make up those clocks are associated with processes related to normal aging in breast. However, most clocks did not exhibit age correlations in breast of women with cancer. This may suggest that epigenetic changes in these samples may have diverged from what is expected during the normal aging process and instead exhibit aberrant age accelerated features, as captured by the Levine and Yang clocks.

The Levine and Yang clock are enriched for or exclusively contain CpGs associated with polycomb-related genes. To examine whether this feature was underlying the observed signal, we created two new clocks out of the Levine clock—one based only on methylation changes seen at CpGs within the original clock that are associated with PcG genes and the other based on changes not co-localizing with PcG targets. We found that the associations for the clock based solely on PcG-related genes showed even stronger associations with breast cancer status than the full Levine clock, while the non-PcG clock did not significantly differentiate cases vs. controls. Polycomb gene protein complexes repress gene expression through epigenetic modification of histone tails and chromatin structure. These complexes play a critical role in cell cycle regulation, DNA damage repair, and cell differentiation [26]. Aberrant methylation of promoters of polycomb group protein target genes is seen in cancer, as well as preneoplastic cells [27, 28]. Tissues with the highest risk of cancer such as breast, prostate, and colon demonstrate the highest levels of aberrant methylation which is correlated with lifetime cancer risk [29]. Our work suggests that age-dependent dysregulation of polycomb-related genes exists in normal breast tissue adjacent to breast tumor, and these changes may precede and herald tumor development. More rapid accumulation of these changes, through accelerated epigenetic aging, may play a role in predisposing individuals to developing breast cancer.

The accelerated epigenetic aging seen in our sample of normal breast tissue that is adjacent to breast tumor suggests that even normal breast tissues in patients with breast cancer have methylation signatures that are closer in resemblance to tumors than controls. We tested this observation by calculating the Mahalanobis distance between cases, controls, and breast tumor samples as reference. Cases had a lower distance to breast tumor samples, suggesting that these samples had a DNA methylation profile more similar to breast tumor samples than controls (*p* = 0.009, Additional file 1: Fig. S5C). There is also a moderate association between age and similarity to tumor DNA methylation profiles (Additional file 1: Fig. S5B).

This signal of accelerated epigenetic aging was only statistically significant in breast tissue and not peripheral blood. This finding suggests that breast tissue is more predictive of incident breast cancer than peripheral blood. This may be due to DNA methylation changes being more predictive of the endogenous hormonal milieu in the breast tissue compared to blood [30]. In addition, while the signal of accelerated epigenetic aging was not picked up in peripheral blood, there is a possibility that it may be seen when cell free DNA is used and this should be explored in future studies [31].

Future prospective studies are needed to determine whether DNA methylation changes in breast tissue precede breast cancer occurrence. A prospective study in peripheral blood showed that accelerated epigenetic age is associated with increased risk of developing breast cancer, however, this study only used 3 clocks (Hannum, Horvath, and Levine) and the signal was low showing hazard ratios ranging from 1.1 to 1.23 [32]. The hazard ratios may be higher when breast tissue is used instead of peripheral blood, or if cell free DNA is used instead of whole blood. The new score utilizing only CpGs that are associated with polycomb-related genes could be a useful clinical tool in the future. A predictive tool is needed for assessing breast cancer risk in women who undergo breast biopsies and are found to have benign lesions but who may be at increased risk for developing breast cancer in the future.

The limitations of this study include small sample size and our study involved mainly luminal breast cancer samples. We cannot extrapolate our findings to all types of breast cancer and we could not evaluate the effect of BRCA mutations due to the small sample size. The strengths of this study include the large size of matched tissues and blood samples, and comparison across multiple epigenetic clocks in order to pinpoint the specific methylation changes associated with cancer.

In conclusion, DNA methylation of normal breast tissue—particularly in CpGs associated with PcG targets—deserves further study as a potential biomarker for breast cancer risk stratification and may lend new insights into mechanisms of breast cancer development.

## Supplementary Information

**Additional file 1.** **Fig. S1.** Consort diagram of breast tissue and peripheral blood samples. **Fig. S2.** Correlations between age and six epigenetic clocks in controls (top row) and cases (bottom row) in breast tissue. **Fig. S3.** Epigenetic clock accelerations in cases (normal breast tissue > 3 cm away from breast tumor in breast cancer patients) and controls (normal breast tissue from healthy individuals) adjusted for confounders (race, smoking, BMI). **Fig. S4.** Correlations between age and epigenetic clocks in controls (top row) and cases (bottom row) in peripheral blood. **Fig. S5.** Using the CpGs in the Levine clock that are associated with polycomb-related genes, we estimated Mahalanobis distance using DNA methylation levels from breast tumor samples as a reference. We then tested whether samples became more tumor like (lower distance) with age in cases (**A**) and controls (**B**). When comparing cases to controls, we found that cases had a DNA methylation profile with a lower Mahalanobis distance suggesting they were more breast tumor like (**C**).

## Data Availability

Upon request.
