# Supplementary material for: Evidence of accelerated epigenetic aging of breast tissues in patients with breast cancer is driven by CpGs associated with polycomb-related genes
Source: Clin Epigenetics. 2022 Feb 24;14:30. doi: 10.1186/s13148-022-01249-z (PMC8876160; doi:10.1186/s13148-022-01249-z)
Supplement: Supplementary file 1 — Additional file 1. Fig. S1. Consort diagram of breast tissue and peripheral blood samples. Fig. S2. Correlations between age and six epigenetic clocks in controls (top row) and cases (bottom row) in breast tissue. Fig. S3. Epigenetic clock accelerations in cases (normal breast tissue > 3 cm away from breast tumor in breast cancer patients) and controls (normal breast tissue from healthy individuals) adjusted for confounders (race, smoking, BMI). Fig. S4. Correlations between age and epigenetic clocks in controls (top row) and cases (bottom row) in peripheral blood. Fig. S5. Using the CpGs in the Levine clock that are associated with polycomb-related genes, we estimated Mahalanobis distance using DNA methylation levels from breast tumor samples as a reference. We then tested whether samples became more tumor like (lower distance) with age in cases (A) and controls (B). When comparing cases to controls, we found that cases had a DNA methylation profile with a lower Mahalanobis distance suggesting they were more breast tumor like (C). [file 13148_2022_1249_MOESM1_ESM.docx]

Supplementary Figure S1


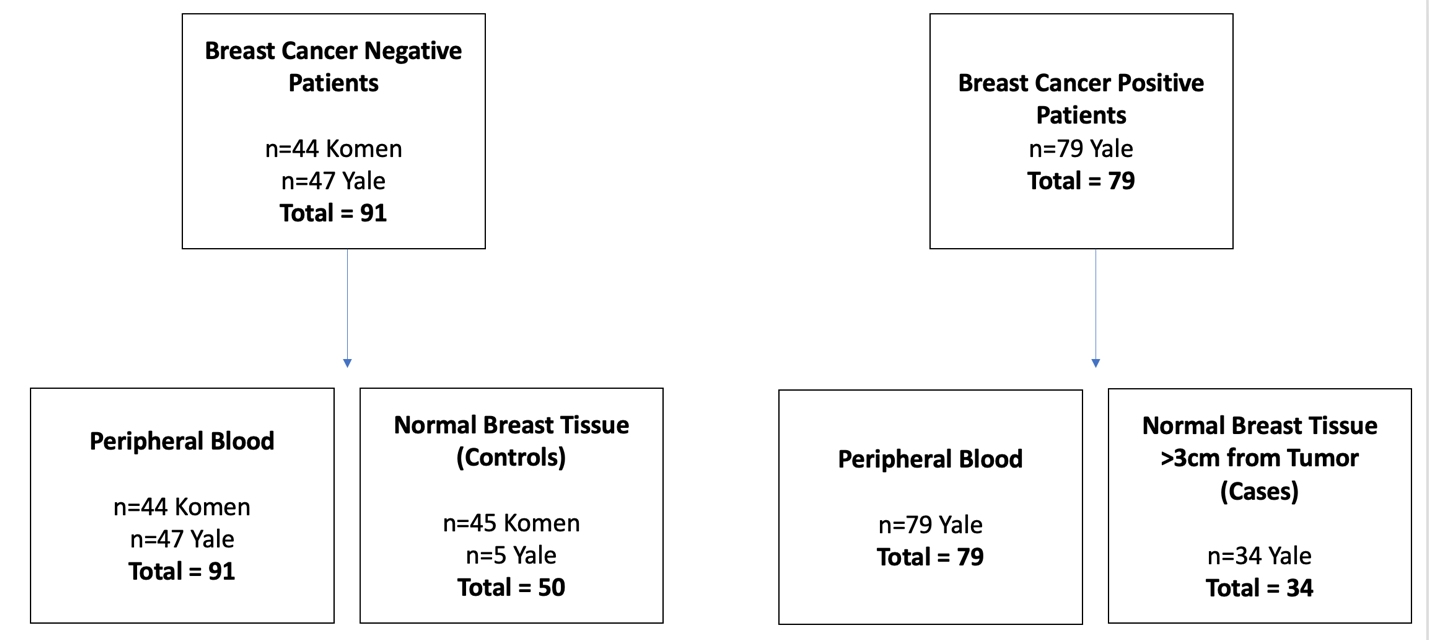


Supplementary Figure S1: Consort diagram of breast tissue and peripheral blood samples.

Supplementary Figure S2


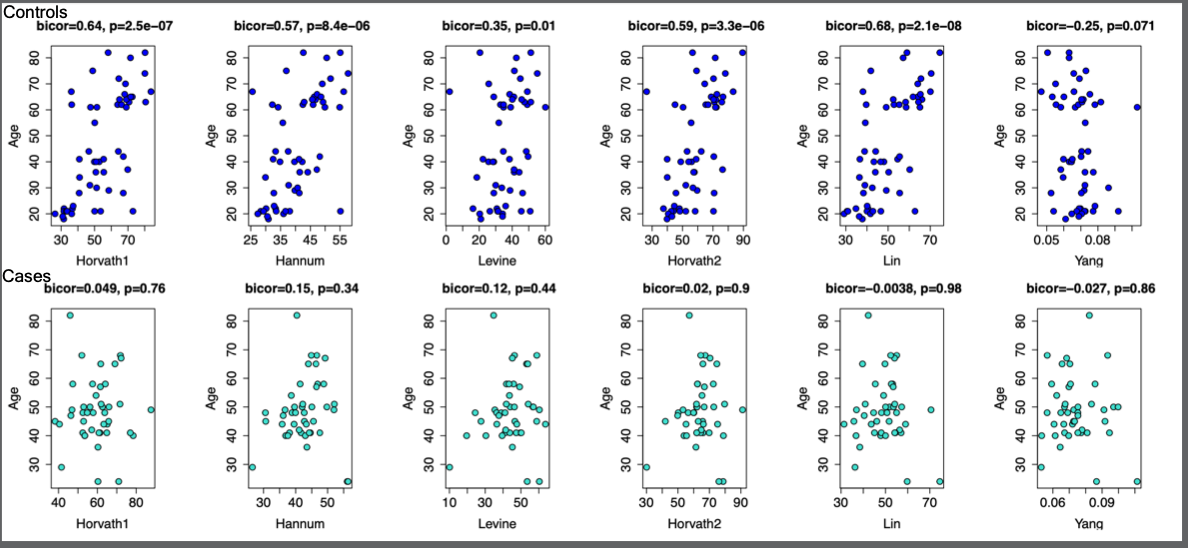


Supplementary Figure S2: Correlations between age and six epigenetic clocks in controls (top row) and cases (bottom row) in breast tissue.

Supplementary Figure S3

Supplementary Figure S3: Epigenetic clock accelerations in cases (normal breast tissue >3cm away from breast tumor in breast cancer patients) and controls (normal breast tissue from healthy individuals) adjusted for confounders (race, smoking, BMI)

Supplementary Figure S4:


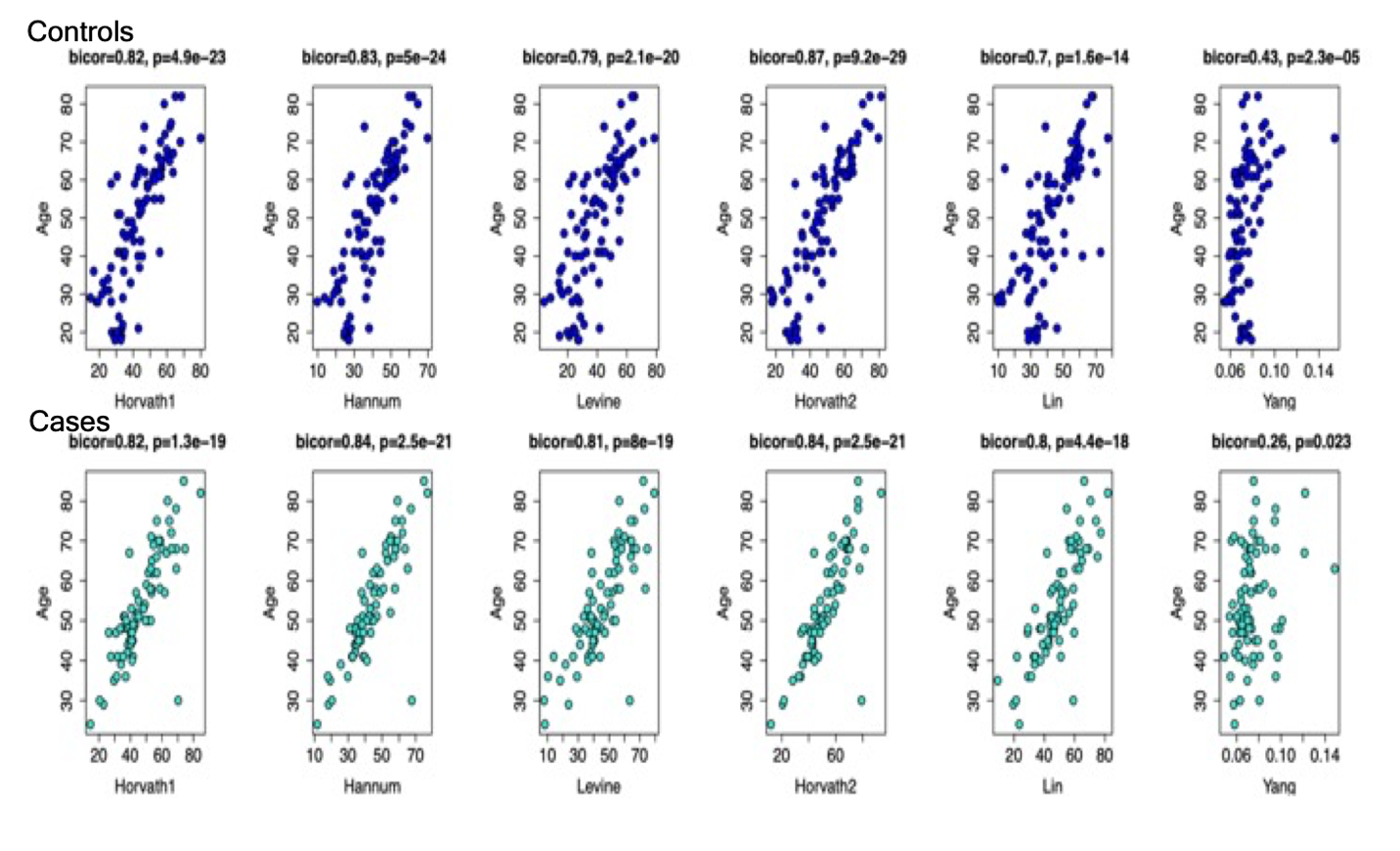


Supplementary Figure S4: Correlations between age and epigenetic clocks in controls (top row) and cases (bottom row) in peripheral blood.

Supplementary Figure S5

**
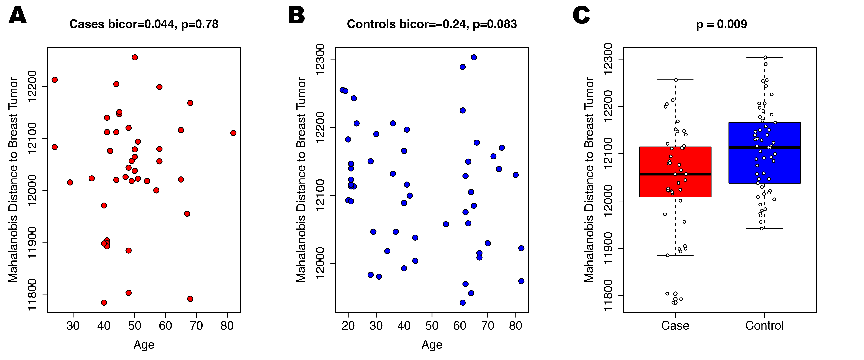
**

Supplementary Figure S5: Using the CpGs in the Levine clock that are associated with polycomb related genes, we estimated Mahalanobis distance using DNA methylation levels from breast tumor samples as a reference. We then tested whether samples became more tumor like (lower distance) with age in cases (A) and controls (B). When comparing cases to controls, we found that cases had a DNA methylation profile with a lower Mahalanobis distance suggesting they were more breast tumor like (C).
